# Acrylamide in Potato Products in Poland from the Perspective of More than 20 Years of Monitoring Study

**DOI:** 10.3390/foods15193485

**Published:** 2026-09-29

**Authors:** Iwona Gielecińska, Hanna Mojska, Jacek Postupolski

**Affiliations:** 1 Department of Food Safety and Health Risk Assessment, National Institute of Public Health NIH—National Research Institute, 00-791 Warsaw, Poland; 2 Department of Nutrition and Nutritive Value of Food, National Institute of Public Health NIH—National Research Institute, 00-791 Warsaw, Poland; hmojska@pzh.gov.pl

**Keywords:** acrylamide, potato products, monitoring, indicative values, benchmark levels

## Abstract

The aim of this study was to analyse the results of nationwide monitoring programmes on acrylamide levels in potato products conducted between 2004 and 2025. This study also assessed the extent to which the legal provisions (10 January 2011, 2013/67/EU; 2017/2158) concerning indicative values and benchmark levels for acrylamide in food affected the levels detected. Data on acrylamide content in 1014 samples representing five groups of potato products were statistically analysed. The highest median acrylamide content was observed in potato crisps (347 µg/kg), followed by potato pancakes (338 µg/kg). The lowest median acrylamide content was found in potato crackers (25 µg/kg). These differences were statistically significant (*p* < 0.005). Over the study period, a significant (*p* < 0.05) reduction in acrylamide content was observed in potato crisps (more than twofold) and in French fries (by almost 40%). The greatest reductions were observed between the initial period (2004–2010) and the period following the implementation of the European Commission recommendations (2011–2017). During this time, acrylamide levels in potato crisps and French fries decreased by more than 60%, indicating the effective implementation of mitigation measures by manufacturers in the potato sector. However, the stabilisation observed in subsequent years suggests, as it appears, that the mitigation measures currently in place no longer enable further reductions in the acrylamide content in potato crisps and French fries.

## 1. Introduction

The potato (*Solanum tuberosum* L.) is the fifth most important crop worldwide, after maize, rice, wheat, and soybeans, with global production exceeding 390 million tonnes of tubers annually [1]. Poland is one of Europe’s leading potato producers, although potato production has been declining since the 1980s, with the rate of decline accelerating at the beginning of the 21st century and continuing to the present. It should be emphasised that, despite a decline in potato production [2] and a fall in total potato consumption [3], consumption of fried potato products in Poland has increased rapidly over the past 30 years. According to household budget surveys, consumption of thermally processed potato products, including French fries and potato crisps, increased almost fourfold, from 0.08 kg per person per month in 2005 to 0.29 kg per person per month in 2021–2022 [Statistics Poland (GUS)—unpublished data]. It is worth noting that fried potato products are currently considered among the most popular snacks consumed worldwide [4].

During the thermal processing of food, new chemical compounds are formed, including process-related contaminants, which may pose a potential health risk. One of the most common process-related contaminants formed in heat-treated, carbohydrate-rich foods is acrylamide (2-propenamide, CAS No. 79-06-1; AA).

In 1994, the International Agency for Research on Cancer (IARC) classified acrylamide as a carcinogen in animals and as a substance probably carcinogenic to humans (Group 2A), stating that while evidence regarding humans was limited, the carcinogenic effect of acrylamide in experimental animals was well documented [5]. Although acrylamide has been shown to have carcinogenic, genotoxic, hepatotoxic, immunotoxic, and adverse reproductive effects in animal models [6,7,8,9,10,11], evidence of its health effects in humans is scarce and inconsistent [12,13]. Nevertheless, in 2024, the IARC priorities working group suggested a reconsideration of the IARC Monographs classification for acrylamide, while also highlighting the high priority of this re-evaluation [14].

In 2002, the Swedish National Food Agency, in collaboration with researchers from Stockholm University, published the first findings indicating high levels of acrylamide in heat-treated, carbohydrate-rich foods, particularly potato and cereal products, as well as coffee and coffee substitutes [15]. In food, AA is formed mainly as a result of the Maillard reaction between free asparagine and reducing sugars, particularly glucose and fructose, at temperatures above 120 °C [16,17]. Small amounts may also be produced as a result of other reactions, including those involving acrolein [18]. The acrylamide content of thermally processed potato products is influenced by numerous factors, ranging from agrotechnical aspects to those associated with the processing and preparation of the final product. Key agronomic factors include potato varieties—which differ in their levels of reducing sugars and asparagine—as well as soil composition and environmental stress [19,20]. Storing potato tubers at low temperatures for a long time also leads to an increase in reducing sugar content and, consequently, an increase in acrylamide levels in fried potato products [21]. The amount of acrylamide formed in potato products is also affected by a number of factors related to the manufacturing process, primarily temperature, duration, and method of heat application; type of oil; slice size; water activity and pH; moisture content; as well as physical parameters such as the surface-to-volume ratio of the food product [22,23].

Immediately following the discovery of acrylamide in food [15], intensive research was undertaken worldwide to develop methods to reduce acrylamide levels in food. It is currently known that suitable potato varieties should be used to prepare potato chips and French fries and that their storage temperature should not exceed 6 °C. Humidity and air circulation should also be adapted to the potato variety to prevent subsequent germination. The production process also involves important steps such as preliminary soaking of potato pieces in water or citric acid solution immediately before frying, as well as lowering the frying temperature and extending the frying time [24].

Recently, Jana et al. [25] pointed out the possibility of using asparaginase to reduce the amino acid content by breaking down asparagine—an amino acid that is a precursor to acrylamide.

In addition, Santiago-Mora et al. [26], using a pulsed electric field (PEF) after peeling potatoes, achieved easier slicing while reducing energy and water consumption. They also found that PEF shortened the frying time by 10% and that the resulting crisps contained 28.9% less acrylamide than the controls [26].

Twenty years after the discovery of acrylamide in food [15], it has become widely recognised that the main dietary sources of this compound are potato products (primarily potato crisps and French fries), processed cereal products (including bread and breakfast cereals, particularly those based on rye, various types of sweet and savoury snacks, cakes, biscuits, and confectionery), as well as coffee and coffee substitutes. Recent studies [27,28] have shown that the estimated mean/median exposure to acrylamide from food worldwide ranges from 0.02 µg/kg body weight/day to 1.9 µg/kg bw/day, reaching a maximum of up to 6.4 µg/kg bw/day. The margins of exposure (MOEs) calculated on this basis are below 10,000 [27], which clearly indicates that this compound poses a risk to public health and that measures should be taken to reduce its dietary intake.

To reduce the acrylamide content in food, the Confederation of the European Food and Drink Industries (CIAA; FoodDrinkEurope since June 2011) published a set of tools, known as the Toolbox (latest version: 2019 [24]), as early as 2005, providing guidance on how to reduce acrylamide levels in industrially produced food.

In May 2007, the European Commission issued Recommendation 2007/331/EC [29] on the Europe-wide monitoring of acrylamide levels in selected food categories, enacted between 2007 and 2009. In the following years, monitoring continued in accordance with Commission Recommendation 2010/307/EU of 2 June 2010 [30], which extended the scope of acrylamide testing to include new food products. Commission Recommendation (EU) 2019/1888 of 8 November 2019 [31] is currently in force; it introduced a non-exhaustive list of foods to be monitored for the presence of acrylamide.

Based on the data collected on acrylamide levels in food [32], the European Commission issued a recommendation in January 2011 on investigations into acrylamide levels in food [33], which established indicative values for this compound for 10 categories of food products. This recommendation, based on successive EFSA reports [27,34], has been amended [35,36] by extending the scope to additional foods and introducing increasingly stringent indicative values and benchmark levels. The currently applicable Commission Regulation (EU) 2017/2158 [36], in addition to benchmark levels, also introduced mitigation measures aimed at reducing acrylamide levels in food, which are to be implemented by manufacturers.

Despite efforts made, acrylamide is still present in food, including potato products. Given the steadily growing popularity and high consumption of thermally processed potato products—specifically crisps and French fries—the aim of this study was to

1. Analyse the results of nationwide monitoring studies on acrylamide levels in selected categories of potato products, carried out over the last 20-plus years;
2. Assess whether the legal provisions establishing indicative values (10 January 2011; 2013/647/EU) [33,35] and benchmark levels, as well as mitigation measures for reducing acrylamide levels in food (2017/2158) [36], were associated with changes in the levels of the compound under investigation in potato products available in Poland.

## 2. Materials and Methods

### 2.1. Data from Monitoring Studies Conducted in Poland Between 2004 and 2025

Data on the acrylamide content in 1014 samples of potato products analysed in monitoring studies conducted in Poland between 2004 and 2025 were statistically analysed. The data were obtained from databases containing the results of food testing conducted as part of official food control and monitoring: the National Food and Nutrition Institute database—results from 2004 to 2018; the National Institute of Public Health—PZH—National Research Institute database—results from 2019 to 2025. The present study included previously reported [37,38,39,40] results on acrylamide levels in potato crisps (n = 186) and French fries (n = 328) from 2004 to 2020. These data were used to conduct a more comprehensive analysis of acrylamide content in potato products based on more than 20 years of monitoring studies conducted in Poland. In addition, other groups of potato products were also included in the study (Table 1). The collected data were checked for validity.

Among potato products, the monitoring studies covered

– Potato crisps and potato crackers;
– French fries (ready-to-eat products and pre-fried semi-finished products);
– Sliced potatoes (e.g., wedges, boat-shaped pieces, rounds, etc.), potato dough products (e.g., balls, cones, etc.), and potato pancakes, purchased as frozen semi-prepared products intended for further preparation at home.

Detailed information on, among other things, the number of samples analysed, the dates of sample collection, and the heat treatment conditions for the semi-finished products is provided in Table 1 and Scheme 1.

The above-mentioned potato product samples were collected throughout the country by official food control authorities (monitoring studies in the years 2004–2025 were conducted with the State Sanitary Inspection) from randomly selected stores and catering establishments.

– 2004–2005—sampling was carried out in accordance with the guidelines and timetable developed by the National Food and Nutrition Institute. For potato crisps, one sample consisted of two retail packs of the product from the same production batch, whereas for French fries from catering establishments, one sample consisted of two portions, with a total weight of 200–300 g;
– 2007–2025—sampling was carried out in accordance with the National Plan for Official Food Control and Monitoring developed by the National Food and Nutrition Institute and the National Institute of Public Health NIH—National Research Institute, based on the guidelines set out in
  (a) European Commission Recommendations (2007/331/EC, 2010/307/EU and 2019/1888/EU) [29,30,31], which specify, among other things, the categories of products to be tested;
  (b) Annex (Part B) to Commission Regulation (EC) No 333/2007 [41], which specifies the sample size and sampling procedure.

The aforementioned control plans were developed taking into account risk assessments, information from the Rapid Alert System for Food and Feed (RASFF), the results of analyses from previous years, and reviews of the current literature.

Acrylamide content was determined in potato product samples at four laboratories that conducted analyses as part of official food control and monitoring:

– 2004–2009—National Reference Laboratory at the National Food and Nutrition Institute; acrylamide was determined in 2004–2007 using gas chromatography–tandem mass spectrometry (GCQ-MS/MS), in accordance with an in-house analytical procedure [42], and in 2008–2009 using liquid chromatography–tandem mass spectrometry (LC-MS/MS), in accordance with an in-house analytical procedure [43];
– 2011–2025—two official food control laboratories designated by the Chief Sanitary Inspector (in Białystok and Gorzów Wielkopolski) and, during the period 2021–2023, the National Reference Laboratory at the National Institute of Public Health—NIH—National Research Institute (NIZP NIH—NRI); acrylamide was determined using the following methods:
  (a) Gas chromatography–mass spectrometry (GC-MS), following derivatisation, in accordance with an in-house analytical procedure (laboratory in Białystok, 2011–2023; laboratory in Gorzów Wielkopolski; [39]);
  (b) Liquid chromatography–tandem mass spectrometry (LC-MS/MS), in accordance with an in-house analytical procedure (NIZP NIH—NRI, [43]) and in accordance with Polish Standard PN-EN 16618:2015 (WSSE in Białystok in the years 2023–2025).

The analytical methods (GC-MS, GCQ-MS/MS, and LC-MS/MS) used by the participating laboratories for acrylamide determination complied with the requirements of Commission Regulations (No 333/2007; 2017/2158) regarding method specificity, limits of detection (LOD) and quantification (LOQ), repeatability, reproducibility, and recovery. Compliance with these performance criteria [36,41] ensured the comparability of the analytical results. All methods used to determine acrylamide content in food have been validated and accredited by the Polish Centre for Accreditation as complying with the requirements of EN ISO 17025 [44].

### 2.2. Analysis of Data on Acrylamide Content in Potato Products from Monitoring Studies Conducted in Poland Between 2004 and 2025

The analysis of the collected data on acrylamide content, conducted as part of this study, comprised

– A comparison of acrylamide levels in various types of potato products analysed in Poland between 2004 and 2025;
– An assessment of the distribution of acrylamide levels in potato crisps and French fries throughout the entire monitoring period;
– An analysis of acrylamide content in potato crisps and French fries, taking into account the period before and after the introduction of the legal provisions establishing indicative values and benchmark levels (European Commission Recommendations: 10 January 2011, 2013/647/EU; Commission Regulation (EU) 2017/2158) [33,35,36];
– A comparison of acrylamide levels in potato crisps in Poland and Europe between 2002 and 2025, taking into account data obtained from published datasets of the European Food Safety Authority (EFSA) and the European Snack Association (ESA) [27,34,45].

### 2.3. Statistical Analysis

Given that the data on acrylamide content in potato products follow a non-parametric distribution, the results are presented as median, minimum, and maximum values, expressed in μg/kg of the ready-to-eat product. For the purposes of comparing acrylamide levels in potato crisps in Poland and Europe (using data compiled by EFSA and ESA), the mean and the 95th percentile were also calculated. Analytical data below the limits of quantification were treated as left-censored observations. To calculate descriptive statistics (mean and median), a middle-bound approach was used, whereby values below the LOD were assigned a value of ½ LOD, while values ranging from LOD to LOQ were assigned a value of ½ LOQ.

The Mann–Whitney U test was used to compare two independent groups. For comparisons of more than two independent groups, the Kruskal–Wallis test was used. Where statistically significant differences were found, post hoc pairwise comparisons were performed using the Mann–Whitney U test. The significance level was adjusted according to the Bonferroni correction to account for multiple testing. A *p*-value of <0.05 was considered statistically significant in all statistical tests. Statistical analyses were performed using Statistica version 13.3 (StatSoft Inc., Tulsa, OK, USA).

## 3. Results

### 3.1. Acrylamide Content in Potato Products

Among the potato products analysed between 2004 and 2025, the highest median acrylamide level was observed in potato crisps (347 μg/kg), followed by potato pancakes and French fries, at 338 and 215 μg/kg, respectively. The lowest acrylamide contents were found in potato crackers (25 μg/kg) and sliced potatoes and potato dough products subjected to heat treatment in accordance with the instructions on the label (79 μg/kg) (Figure 1). These differences were statistically significant (*p* < 0.005). It should be noted that only potato crisps and French fries were tested over the more than 20-year period covered by the monitoring studies. The other potato products were tested only sporadically. Baked potato pieces and potato dough products were tested in only one year, while potato crackers in two non-consecutive years and potato pancakes in three non-consecutive years. In all five categories of potato products tested, the median AA content did not exceed the indicative values set out in the European Commission Recommendations (of 10 January 2011 and 8 November 2013) or the benchmark levels established in Regulation (EU) 2017/2158. Detailed analysis of the data showed that more than 97% of the potato product samples tested during the period when these legal provisions were in force (2011–2025) met the criteria specified in the aforementioned documents (Figure 1 and Figure 2). Exceedances of the indicative values and benchmark levels for this compound were observed only sporadically in individual samples of potato product. This applied to 2.9% of potato snacks (13 samples of potato crisps and 1 sample of potato crackers), 1.9% of fried potato products (7 samples of French fries), and 4.2% of potato pancakes (2 samples) (Figure 1 and Figure 2). However, no exceedances of the indicative values or benchmark levels for acrylamide were observed among the baked sliced potatoes (e.g., wedges, boat-shaped pieces, rounds, etc.) and potato dough products (e.g., balls, cones, etc.) (Figure 1). Before the introduction of the legal provisions on acrylamide levels in food in 2011, individual samples of potato crisps and French fries contained acrylamide at levels considerably higher than the indicative values and benchmark levels that were subsequently established, ranging from 1346 to 3647 μg/kg and 1144 to 2175 μg/kg, respectively.

A further detailed statistical analysis was carried out solely for the categories of potato crisps and French fries, as acrylamide levels were determined only in these products throughout the entire monitoring period of more than 20 years.

### 3.2. Analysis of Acrylamide Content in Potato Crisps and French Fries over More than 20 Years of Monitoring Studies

A decline in the median acrylamide content was observed in both potato product categories over more than 20 years of monitoring period (Figure 3 and Figure 4). In potato crisps, the median concentration of acrylamide investigation decreased by more than twofold, from 754 μg/kg in 2004 to 358 μg/kg in 2025, representing a statistically significant reduction (*p* < 0.00001) (Figure 3). In French fries, the reduction in median acrylamide content between the beginning and the end of the study period was smaller but also statistically significant (*p* < 0.05), at almost 40%, from 307 μg/kg to 191 μg/kg (Figure 4).

A detailed analysis of data collected over more than 20 years of monitoring studies showed that the greatest decrease in median acrylamide content, of approximately fourfold, occurred between the initial study period, before the introduction of European Union legislation on acrylamide monitoring in food, and 2014 for French fries and 2015 for potato crisps. During this period, the concentration of acrylamide investigation fell from 307 μg/kg to 79 μg/kg (French fries) and from 754 μg/kg to 180 μg/kg (potato crisps). In subsequent years, following the period of the greatest reduction in acrylamide content, the median content of the compound under investigation in French fries remained relatively stable, ranging from 154 to 195 μg/kg, apart from a single decrease in 2017 to 82 μg/kg, a level similar to that observed in 2014 (Figure 4). However, as shown in Figure 3, the median acrylamide content of potato crisps showed slight short-term fluctuations in both directions in subsequent years (2016–2025), ranging from 253 μg/kg to 439 μg/kg.

It is also worth noting that a comparison of the minimum and maximum acrylamide levels at the initial and final stages of the study indicates a decrease in acrylamide content across the product categories analysed. In the potato crisps category, the minimum level of acrylamide in 2004 was 352 μg/kg and the maximum was 3647 μg/kg, whereas in 2025, the corresponding values were 88 μg/kg and 804 μg/kg, respectively (Figure 3). By comparison, in the French fries category (Figure 4), the corresponding values were 155 and 799 μg/kg (2005), and <8 μg/kg (LOD) and 480 μg/kg (2022), respectively.

The above data show that, over the 22 years of monitoring in Poland, there was a reduction not only in the calculated median acrylamide content of the products tested but also in the minimum and maximum values determined in individual samples (Figure 3 and Figure 4). The decrease in the lowest recorded values was fourfold for potato crisps and approximately twentyfold for the French fries group. For the highest recorded values, acrylamide content decreased by more than 4.5-fold in potato crisps and by almost twofold in French fries.

Figure 5 shows a comparison of acrylamide content in potato crisps in Poland and Europe. The data shown are derived from monitoring studies conducted in Poland between 2004 and 2025, from data collected by European Food Safety Authority (EFSA) data in European Union countries since 2007, and from data reported by the European Snacks Association (ESA) for the period 2002–2019. An analysis of the above results showed that, both in Poland and in other European countries (based ESA data), the mean acrylamide content in potato crisps showed a clear decrease between the initial data collection period and 2011 (ESA) and 2015 (Poland). After this period, the average concentration of acrylamide stabilised, with slight short-term upward and downward fluctuations. A similar decrease can be seen in the data for the 95th percentile value. The pattern was different for the EFSA data derived from European monitoring studies. During the initial period (2007–2010), the mean acrylamide content in potato crisps was followed in subsequent years by alternating marked decreases (with minima in 2013 and 2017–2018) and increases (with a maximum in 2015). For high acrylamide levels (P.95), following an initial increase (2007–2009), an overall decline was observed in subsequent years. It should be noted that, although in 2007, the data from all three sources were identical (mean value) or similar (95th percentile), the acrylamide content in potato crisps reported in the European monitoring studies compiled by EFSA was higher, for both the mean value and the 95th percentile throughout the subsequent period of more than 10 years, than the corresponding values reported in Poland and by the ESA (Figure 5).

### 3.3. A Comparison of Acrylamide Content in Potato Crisps and French Fries Analysed in Poland Before and After the Introduction of Legislation Establishing Indicative Values and Benchmark Levels for This Compound in Food

The more than 20-year monitoring period in Poland was divided into three time periods, corresponding to the periods before and after the introduction of legislation establishing indicative values, benchmark levels, and mitigation measures for acrylamide content in food. Period 1 (2004–2010) began two years after reports that food could be a source of acrylamide first emerged. During this period, there were no legal requirements regarding acrylamide content in food, either in Poland or in the European Union. Period 2 (2011–2017) followed the introduction of the Recommendations (of 10 January 2011 and 8 November 2013), which established indicative values for acrylamide (potato crisps made from fresh potatoes and potato dough—1000 μg/kg; ready-to-eat French fries—600 μg/kg), while Period 3 (2018–2025) followed the establishment of benchmark levels under Regulation (EU) 2017/2158, which were lower than the indicative values for acrylamide in selected food categories (potato crisps—750 μg/kg; French fries—500 μg/kg).

Statistical analysis of the collected data revealed statistically significant differences between the study periods (potato crisps—H [2, N = 535] = 69.08145, *p* < 0.00001; French fries—H [2, N = 387] = 85.9923, *p* < 0.0001). The highest median acrylamide content was recorded in potato products during the first study period (*p* < 0.005). In both groups, these values were more than 2.5 times higher than in the second period and twice as high as in the third period (Figure 6).

Figure 7 and Figure 8 show the distribution of potato product samples by acrylamide content across the three study periods. In both potato product groups, the introduction of regulations on acrylamide content was followed by a marked increase in the proportion of samples with lower acrylamide content. In the potato crisps category, the proportion of samples not exceeding 1000 μg/kg (the 2011 indicative value) increased from 72.9% in the first period to 98.9% in the second period (Figure 7). In the French fries category, when the 2011 indicative value (600 μg/kg) is also included in the analysis, the corresponding values are 78.5% and 98.4%, respectively (Figure 8).

Even greater differences in the number of samples were found when values equal to half of the indicative values were used for the assessment. In the potato crisps category, acrylamide content not exceeding 500 μg/kg was found in 35.6% of the samples tested before the introduction of the aforementioned legislation, and in 83.2% (2011–2017) and 78.0% (2018–2025) of the samples thereafter (Figure 8). The situation was similar for French fries. Between 2005 and 2010, acrylamide levels of up to 300 μg/kg were found in 29.1% of the samples tested, whereas following the introduction of the Recommendations (Period 2) and Regulation (EU) 2017/2158 (Period 3), which established indicative values and benchmark levels for acrylamide, this proportion increased to over 70% (Figure 8).

Analysis of the data showed changes in not only the mean acrylamide content in potato products but also the maximum values. During the first study period, the maximum concentration of the compound under investigation was 3647 μg/kg in potato crisps and 2175 μg/kg in French fries. Following the introduction of the legislation establishing indicative values and benchmark levels for acrylamide in food, the maximum values decreased by twofold in potato crisps and by more than 3.5-fold in French fries.

## 4. Discussion

In Poland, monitoring studies of acrylamide content began as early as 2004, two years after reports emerged that acrylamide was formed during the thermal processing of food [39]. Given that, at that time, the highest acrylamide levels were found in processed potato products and that potatoes are an essential part of the Polish diet, the initial focus was primarily on potato crisps and French fries. These two product categories were monitored throughout the entire monitoring period of more than 20 years in Poland. This was also due to the fact that only these two groups of potato products had been covered by European monitoring studies since 2007. It is worth noting that in Poland, monitoring studies have also included other potato products popular among consumers, such as baked potato pieces and potato dough products (2023), potato snacks (2015 and 2021), and potato pancakes (2014, 2018, and 2025).

### 4.1. Potato Snacks

#### 4.1.1. Potato Crisps

Among the potato products analysed, potato crisps had the highest, statistically significant (*p* < 0.005) acrylamide content. Over the 2004–2025 monitoring period in Poland, the median concentration of this compound was 347 μg/kg, ranging from below the limit of detection (LOD = 8 μg/kg) to over 3500 μg/kg. Results similar to the average concentration of acrylamide found in our study were reported in studies conducted in the USA between 2011 and 2015 [46], in Belgium between 2008 and 2013 [47], and in North Macedonia [48] and Denmark [49] in 2018. Similar or slightly lower mean acrylamide content in various commercial brands of potato crisps was also reported in Canadian studies in 2009–2016 [50,51]. However, these values were comparable to the average concentration of the compound under investigation obtained in monitoring studies conducted in Poland in 2012, 2017–2018, 2022, and 2024. The median acrylamide content in potato crisps obtained in our studies over more than 20 years was also similar to the mean content in Europe, calculated on the basis of pan-European monitoring studies conducted between 2007 and 2012 [27], as well as to the results obtained in Romania [52].

Higher mean acrylamide content than the median observed in our study was reported for potato crisps analysed in monitoring studies in Switzerland between 2007 and 2009 [53] and in the Czech Republic in 2013 [54], as well as in studies conducted by the European Snacks Association (ESA) between 2010 and 2019 [45], and in earlier studies by Claeys et al. [47] and Abt et al. [46], conducted shortly after the discovery of acrylamide in processed foods.

Markedly higher mean or median acrylamide contents, averaging 500–600 μg/kg, were reported by Normandin et al. [55] in Canada; Mesias and Morales [56], and Mesias et al. [57] in Spain; Cressey et al. [58] in New Zealand in 2011; Orioan et al. [59] in Romania; Pugajeva et al. [60] in Latvia; Schneeweiss [61] in the Czech Republic (monitoring studies over the last 4 years); and Lee et al. [62] in Korea. Similar values were obtained by Wenzl and Anklam [63] in their analysis of the acrylamide content in potato crisps tested between 2002 and 2006 in the European Union, mainly in Germany (data from official food controls and private laboratories), as well as in data reported by the European Food Safety Authority (EFSA) from pan-European monitoring studies from 2011 to 2016 [45]. These values were also similar to the median acrylamide content recorded in Poland during the early years (2007–2009) of its participation in pan-European monitoring studies.

Mean acrylamide contents almost or more than twice as high as the median content recorded in potato crisps analysed in Poland between 2004 and 2025 (347 μg/kg) were reported by Arribas-Lorenzo and Morales [64] in Spain, Hamlet et al. [65,66] in the United Kingdom, Pacetti et al. [67] in Colombia, Sirot et al. [68] in France, Janković et al. [69], Korićanac et al. [70] in Serbia, and Turalić et al. [71] in Bosnia and Herzegovina in 2024, as well as Mesias et al. [72] in Spain. Similar values were also observed in the early years (2002–2005) of the ESA’s research [45] and in Czech monitoring studies (2005 and 2009–2021) [54,61].

Even higher mean and/or median acrylamide contents, exceeding 1000 μg/kg and reaching as much as 1600 μg/kg, were reported for potato crisps from New Zealand [73], Syria [74], India [75], the Czech Republic (2004 and 2006–2008) [54], and Italy [76], as well as for certain commercial brands of potato crisps tested in Lebanon [77] and Canada in 2009–2016 [51]. These figures were similar to the maximum values recorded in Poland during the first five years of the monitoring programme. Analysis of the maximum acrylamide content in potato crisps in Poland over the last 22 years showed that the values obtained were similar to the 90th and 95th percentile values and maximum levels reported in other European countries [27,45,47,49,52,56,57,59,69,70,72,76] and at various centres around the world [46,51,55,58].

#### 4.1.2. Potato Crackers

Another type of potato snack that is popular in Poland, although less so than potato crisps, is potato crackers. The median acrylamide content in these products was almost 14 times lower than in potato crisps. This may be due to differences in the ingredients used in the snacks and the heat-treatment methods applied. Potato crisps are thin slices of raw potatoes or thin rounds made from potato dough that are deep-fried. In the case of potato crackers, however, the main raw material is usually potato starch, although potato flakes, potato granules, or dried potato may also be used. These and other ingredients are used to produce a mixture, which is usually shaped into discs and then subjected to an extrusion process.

Among potato crackers analysed in Poland, the median acrylamide content was 25 μg/kg, with values ranging from below 8 μg/kg (LOD) to 383 μg/kg; only one sample reached 1122 μg/kg. The acrylamide content in these products was strongly associated with the content of potato ingredients. The higher the proportion of these ingredients, the higher the acrylamide content of the potato crackers under investigation.

The acrylamide levels in potato crackers obtained in monitoring studies conducted in 2015 and 2021 were similar to these reported in our earlier studies, in which the content of the component under investigation ranged from 130 to 216 μg/kg of potato crackers (Gielecińska & Mojska, 2010–unpublished data), as well as to the results of pan-European monitoring studies conducted between 2007 and 2012 [27]. A similar content of acrylamide was also reported by Tsutsumiuchi et al. [78]. It should be noted, however, that the concentration found by these authors in a single sample was more than three times higher than the maximum concentration determined in our monitoring studies. Korićanac et al. [70] reported values more than 20-fold higher in Serbia; however, as the authors themselves stated, the group of potato snack products included various products made from potato dough, including snacks, potato crisps, and potato crackers. A potato crisp sample analysed by Mihai et al. [52] in Romania also had a high acrylamide content of 1500 μg/kg. In contrast, other Japanese studies [79] reported significantly lower acrylamide levels in potato crackers, not exceeding 70 μg/kg.

### 4.2. Fried/Baked Sliced Potatoes and Potato Dough Products

Another group of potato products covered by the monitoring studies in Poland comprised fried or baked sliced potatoes and potato dough products. This group included French fries and other cut potato products, such as wedges, slices, and rounds, which were either collected as ready-to-eat products from bars and restaurants or purchased from shops as frozen semi-finished products and then prepared according to the instructions on the label. Products made from potato dough included, for example, balls and croquettes, as well as potato pancakes sold as semi-finished products for further heat treatment in catering establishments or at home.

#### 4.2.1. French Fries

The acrylamide content in French fries sampled on the Polish market between 2005 and 2022 had a median value of 215 μg/kg, ranging from below 8 μg/kg (LOD) to over 2000 μg/kg. A similar median content of acrylamide was reported for French fries analysed in Belgium between 2008 and 2013 [47], while slightly higher levels were reported in Canadian studies of oven-baked chips [55], Spanish studies of chips collected from restaurants [80], and Italian studies of deep-fried chips [81]. Even higher average or median acrylamide contents, ranging from 300 to 400 μg/kg, were reported in French fries analysed as part of pan-European monitoring studies [27], as well as in samples analysed in Switzerland [53], New Zealand [58], and the USA [46]. Acrylamide levels approaching or exceeding three times the median content recorded in our monitoring studies conducted over more than 20 years were reported by Dimitrieska-Stojkovik et al. [48] in North Macedonia and Bajić et al. [82] in Serbia in 2024, Sirot et al. [68] in France, and Normandin et al. [55] in Canada, all of whom analysed deep-fried French fries.

By contrast, Biedermann et al. [53] reported a lower acrylamide content, 100–150 μg/kg, in French fries prepared from semi-finished products; Mesías et al. [83] in French fries collected from school canteens in Spain; Mihai et al. [52] in French fries collected from Romanian fast-food restaurants; Hossain et al. [51] in Canadian fast-food restaurants; and Claeys et al. [47] in Belgium. It should be noted that, with the exception of the Romanian studies, despite a lower mean or median acrylamide content reported in the other studies cited, the maximum level was similar to, and usually higher than, the results of our monitoring studies. In Poland, the maximum acrylamide content in French fries collected from bars and restaurants and prepared from pre-fried frozen semi-finished products was 866 μg/kg. Significantly higher values were found only in 2007 in three samples of French fries prepared from raw potatoes, with acrylamide contents of 1144, 2133, and 2175 μg/kg. Similar maximum values were found in studies conducted in New Zealand [58], Belgium [47], Canada [55], and Spain [83]. It is also worth noting that significant variation in results was observed within the group, both in Polish monitoring studies and in the majority of the cited studies; that is, some products were characterised by very low values, sometimes even below the limit of quantification, whilst others had very high levels, exceeding 1000 μg/kg or 2000 μg/kg, or even reaching 4000 μg/kg.

#### 4.2.2. Sliced Potatoes and Products Made from Potato Dough

In Poland, monitoring studies conducted in 2023 covered not only French fries but also sliced potatoes (e.g., wedges, rounds, etc.) as well as products made from potato dough, such as balls, cones, etc., which were purchased as frozen semi-finished products and then prepared according to the manufacturer’s instructions on the label. These products had a median acrylamide content almost three times lower than that of the French fries discussed above (79 vs. 215 μg/kg). This lower content may be due to differences in the raw materials used, the type of heat treatment applied during the production of the aforementioned potato products, or the surface area-to-mass ratio of the product. These parameters can have a significant impact on the acrylamide content of the finished product. Thus, French fries prepared from raw potatoes are generally characterised by a higher content of acrylamide than French fries prepared from semi-finished products, whose production involves the use of available mitigation measures to reduce the acrylamide content in the final product [27,51,83,84,85]. The manufacturer can, among other measures, select a more suitable potato variety for French fries and store it at an appropriate temperature—apply various pretreatment steps, such as blanching—aimed at partially removing precursors, mainly reducing sugars, and use appropriate parameters for the initial thermal treatment, including time, temperature, as well as the colour of the final product. During thermal processing, deep-frying usually results in higher acrylamide content in French fries than baking (e.g., in a convection oven or air fryer) or prepared in a microwave oven [27,55,81,86]. In our research, the “French fries” category, included both products prepared from raw potatoes and frozen, pre-fried semi-finished products, with deep-frying being the main method of heat treatment, both in bars and restaurants and under laboratory conditions. The potato pieces, however, were pre-fried products that were subsequently baked, in an air fryer or convection oven, in accordance with the manufacturer’s instructions on the packaging. Furthermore, the potato pieces had a larger surface-area-to-mass ratio than French fries, which may also have affected the acrylamide content in the finished product [23]. It is well established that acrylamide forms primarily at the surface of the product [87,88,89]. Potato pieces have a higher surface-to-mass ratio than French fries, particularly the thinner and crinkle-cut varieties, which may also contribute to the significant (*p* < 0.005) difference in acrylamide content between the two products. Furthermore, some samples in this category consisted of products made from potato dough. Potatoes are also their main ingredient but are used in purée form, from which products of various sizes and shapes are moulded. It is reasonable to assume that using the same raw material but in a different form (a potato piece vs. purée) may also affect the acrylamide content in the finished product. The results of our research appear to support this assumption. The acrylamide content in potato pieces and potato dough products averaged 162 and 103 μg/kg, respectively. However, the difference observed was not statistically significant, which may be due to the small number of samples tested.

In monitoring studies conducted in Poland in 2011, the median acrylamide content in sliced potatoes and products made from potato dough baked according to the instructions on the product label was 4- to 9-fold lower than the values reported by Michalak et al. [90]. These products, subjected to various types of heat treatment, had acrylamide contents ranging from 341 to 698 μg/kg. However, it should be emphasised that the commercial semi-finished products analysed by Michalak et al. [90] already exhibited substantially higher acrylamide concentrations, with levels nearly 50% higher in potato cubes and potato wedges and more than fourfold higher in potato noisettes, compared with the average concentrations obtained in our monitoring study for baked sliced potato and formed products made from potato dough. Notably, the study by Michalak et al. [90] was conducted before the implementation of the European Commission Recommendation (10 January 2011) establishing indicative values for acrylamide in food, which may partly explain the higher acrylamide concentrations reported.

#### 4.2.3. Potato Pancakes

Another group of products made from potato dough comprises potato pancakes. The median acrylamide content in these products was more than three times higher than in the potato dough products, discussed above, such as balls, croquettes, and similar products. This may be due to differences in the ratios of baked surface area to product mass, as mentioned earlier, with potato pancakes having a large surface area and balls, croquettes, etc., having a much smaller one. Furthermore, this category included both products made from frozen, pre-fried semi-finished products (in catering establishments and under laboratory conditions) and potato pancakes prepared from raw potatoes in bars and restaurants.

The median acrylamide content in potato pancakes (338 µg/kg) from monitoring studies in Poland was lower than the values reported in the literature. The potato pancakes analysed in the European Union between 2002 and 2006, primarily in Germany, showed the most similar median AA content, though it was approximately 10% higher [63]. In a study by Michalak et al. [90], potato pancakes prepared from pre-fried semi-finished products purchased on the Polish market had an average acrylamide content of 529 μg/kg, ranging, depending on the heat treatment applied, from 422 to 694 μg/kg. Comparable results were also obtained in other studies conducted in Germany [91,92], while slightly higher values were reported for rösti pancakes, popular in Switzerland [93]. It should be noted that the above mean or median values were close to the 95th percentile value obtained in our monitoring studies. In contrast, fried potato pancakes prepared at home from raw potatoes had an acrylamide content almost three times higher than that found in the monitoring studies [94]. These data indicate that these are potato products for which specific measures to mitigate acrylamide levels, such as the addition of asparaginase, should be implemented [36].

When comparing acrylamide content across all groups of potato products, it is important to note that, unlike our long-term studies, the results of most of the cited studies are based on one-off analyses of acrylamide levels conducted at a specific point in time and, in most cases, in a single town or region, involving only a small number of samples. Furthermore, these studies differ in the way they present their results: mean, median, 95th percentile, or simply the range of results. This makes it difficult to compare the results across studies. It should be emphasised that our analysis covered the results obtained in monitoring studies conducted in Poland between 2004 and 2025, including 535 samples of potato crisps and 387 samples of French fries. In the case of potato pancakes, 48 samples were analysed in 2014, 2018, and 2025, while 13 samples of potato crackers and 31 samples of potato pieces were analysed in 2015 and 2021 (potato crackers), and 2023 (potato pieces or products made from potato dough), respectively.

### 4.3. Analysis of Acrylamide Content in Potato Crisps and French Fries in Monitoring Studies Conducted Between 2004 and 2025

#### 4.3.1. Potato Crisps

Over more than 20 years of monitoring studies conducted in Poland, a significant (*p* < 0.0001) reduction in acrylamide levels was observed in both categories of potato products. The reduction in the acrylamide content in potato crisps was over 64%. A decrease in average acrylamide content comparable to that observed in our studies between 2004 and 2025 was recorded in monitoring studies conducted in the Czech Republic during the same period [54,61]. On the other hand, Powers et al. [45], analysing data collected by the European Snack Association (ESA) between 2002 and 2019, and Mesias et al. [72], in Spain between 2004 and 2025, found slightly smaller reductions. According to ESA research, acrylamide levels fell by over 51% between 2002 and 2019, whilst Spanish monitoring studies showed a 40% reduction. A significantly smaller reduction in the acrylamide content of potato crisps was reported in pan-European monitoring studies conducted between 2011 and 2018 [45]. The observed decrease of just under 20% may be related to the fact that the first year included in the comparison was 2011, when the European Commission Recommendation on indicative values for acrylamide in food was already in force. It appears that the potato sector was simultaneously and effectively implementing the mitigation strategies published in the Toolbox (latest version: 2019 [24]). A similar reduction of over 20% in the acrylamide content of potato crisps was also reported by Abt et al. [46] in studies conducted in the United States. In this case, such a slight reduction may be due to the fact that the authors compared findings from two shorter periods: 2002–2006 and 2011–2015. Similarly, Canadian studies conducted between 2009 and 2016 found a reduction in acrylamide content in potato crisps, but only for one commercial brand. In other cases, slight changes in either direction were observed [51].

As in our study, the above-mentioned authors observed a decrease in acrylamide content not only in the calculated mean or median values but also in the highest recorded values (maximum content) or the values reported for the 90th or 95th percentile.

#### 4.3.2. French Fries

Compared with potato crisps, a significantly smaller reduction in acrylamide content was observed for French fries over more than 20 years of monitoring studies conducted in Poland. The difference in content between the beginning (2005) and the end (2022) of the period was approximately 40%. This applied to both the median and maximum values, 307 and 799 μg/kg (French fries, 2005), and 754 and 3647 μg/kg (potato crisps, 2004), respectively. A reduction in acrylamide content in French fries was also observed in Canadian studies conducted in 2009 and 2014. Hossain et al. [51] also found a reduction in acrylamide content in ready-to-eat French fries sampled in 2009 and 2016 from two of the three fast-food chains operating on the Canadian market. The reduction in acrylamide content in French fries may be due to the fact that currently available methods for reducing this compound in fries do not allow for achieving lower acrylamide levels in such products. Furthermore, our research indicates that the indicative value and benchmark level specified in the European Commission Recommendations and Commission Regulation (600 µg/kg, 500 µg/kg) are not exceeded in the majority of samples; consequently, there may be a lack of incentive to seek or implement new methods for reducing acrylamide content in French fries.

In both categories of potato products examined, the greatest decrease in acrylamide content was observed between the initial study period and the year in which the first European Commission Recommendation setting indicative values for 10 food categories that may be sources of acrylamide was published (10 January 2011). A similar pattern for potato crisps was also reported by Powers et al. [45] in European Snack Association (ESA) surveys and by Mesias et al. [72] in Spanish monitoring studies. In the study by Powers et al. [45], this occurred in 2011, as was the case in Poland, whereas in Spain, it occurred in 2014. This difference may be due to the fact that Mesias et al. [72] analysed changes based on monitoring data from five selected years (2004, 2008, 2014, 2019, and 2025). In monitoring studies conducted in the Czech Republic, the greatest reduction in acrylamide content in potato crisps was observed between 2004 and 2008 [54].

A significant decrease in acrylamide levels observed over more than 20 years of monitoring in Poland across both categories of potato products appears to indicate the effective implementation of acrylamide reduction measures by producers in the potato-processing sector. However, the observational nature of the data does not allow causal conclusions to be drawn. It should be noted that, as early as 2005, the Confederation of the EU Food and Drink Industries (now FoodDrinkEurope) developed guidelines (the so-called “Toolbox”) to help producers reduce acrylamide levels in food. Since April 2018, food producers, in accordance with Commission Regulation (EU) 2017/2158, have been required to apply mitigation measures aimed at reducing the presence of this compound in finished food products. It appears that measures taken by producers, including the use of appropriate potato varieties, blanching the raw material to remove reducing sugars, and optimisation of thermal processing conditions (including processing time, temperature, and the colour of the final product), have contributed to an effective reduction in acrylamide levels. This is confirmed by the results of our monitoring studies. In Poland, the acrylamide content of potato crisps and French fries decreased by more than 60% between the initial period (2004–2010) and the period following the introduction of the European Commission recommendations (2011–2017). At the same time, the role of the recommendations (10 January 2011, 2013/647/EU), as well as Commission Regulation (EU) 2017/2158, should not be overlooked. These measures initially introduced indicative values, which evolved over the years, and subsequently benchmark levels. Although these values did not constitute legally binding maximum levels, exceeding them indicated the need to carry out further investigations into the production and processing methods used by food producers. In our opinion, these legislative measures may also have contributed to a further reduction in acrylamide levels in food. The lack of a significant change in acrylamide content in French fries between the second (2011–2017) and third (2018–2022) study periods appears to indicate that a stabilisation has been reached in acrylamide levels in potato products. It seems that, with the current production techniques and mitigation measures applied to these products, producers have reduced acrylamide levels in finished products to the lowest achievable levels. This is clearly evident in studies conducted after 2011. These findings are also supported by studies conducted in other countries [45,51,54,61,72]. This may indicate that the mitigation measures currently in place no longer allow for further reduction in acrylamide content in potato products. At the same time, the significant increase in acrylamide content observed in potato crisps tested during 2018–2025 is surprising. However, this may be related to the larger number of samples analysed in this period than in the two previous periods (387 vs. 148).

To sum up, it should be noted that the monitoring programme described is unique in terms of its duration, as it makes it possible to compare the acrylamide content of potato products available on the market before and after the entry into force of legislation introducing indicative values, benchmark levels, and mitigation measures for acrylamide in food, rather than merely presenting the content of the compound under investigation in potato products over the years.

### 4.4. Strengths and Limitations

We acknowledge several limitations of this study, including the fact that the analytical determinations performed throughout the study period were conducted by four different laboratories using several analytical methods (GC-MS, GCQ-MS/MS, and LC-MS/MS). However, all methods were validated, accredited in accordance with EN ISO/IEC 17025, and complied with the criteria established by the European Commission (Commission Regulation (EC) 333/2007 and Commission Regulation (EU) 2017/2158). Although differences between methods in terms of instrumentation, limits of detection and quantification, recovery, and measurement uncertainty cannot be entirely excluded, all methods were considered fit for their intended purpose, namely official food control and food monitoring. Therefore, the potential impact of methodological changes on the observed temporal decrease in acrylamide concentrations was considered limited compared with the substantial variability in acrylamide levels observed throughout the study period.

A major strength of this study is the exceptionally long monitoring period, spanning 22 years for potato crisps and French fries. In addition, more than 900 samples were analysed, providing a robust basis for assessing long-term changes in acrylamide occurrence. Samples were collected throughout the country by official food control inspectors in accordance with established sampling plans, ensuring broad geographical coverage and improving the representativeness of the dataset. Sampling plans for acrylamide monitoring in food were developed annually, in line with the recommendations of the European Commission (2007/331/EC, 2010/307/EU, and 2019/1888/EU) and relevant legislation (Regulation (EC) No. 333/2007). Furthermore, to our knowledge, this is the first study to evaluate whether the establishment of indicative values (in 2011) and benchmark levels (in 2017) for acrylamide in food, together with the implementation of mitigation measures aimed at reducing its formation, may have contributed to the observed reduction in acrylamide concentrations in potato products over time.

## 5. Conclusions

An analysis of the results of monitoring studies on acrylamide content in potato products, conducted in Poland between 2004 and 2025, showed a significant decrease in the content of this compound in the potato crisps and French fries groups between the beginning and the end of the study period for each group. Potato crisps still have the highest acrylamide content. A relatively high acrylamide content was also observed in potato pancakes. Our results indicate the need to strengthen the mitigation measures implemented by manufacturers of this product group. It should be emphasised, however, that during the period in which these legal provisions were in force (2011–2025), the acrylamide content did not exceed the specified indicative values or benchmark levels in more than 97% of the potato product samples analysed. Exceedances of these values were found in individual samples of potato crisps, potato crackers, French fries, and potato dough products, indicating the need for continued monitoring not only of potato crisps and French fries but also of other groups of potato products.

The greatest reduction in acrylamide content in potato crisps and French fries was observed between the initial period (2004–2010) and the period following the entry into force of the first European Commission Recommendation setting indicative values for selected food categories. The reduction observed indicates that manufacturers in this sector are implementing effective acrylamide mitigation measures described in the Toolbox. The introduction of legislation establishing indicative values and benchmark levels appears to have supported these measures. The mitigation measures currently in place appear to be insufficient to achieve further reductions in acrylamide content in potato crisps and French fries. Given the confirmed adverse effects of acrylamide on human health, including its carcinogenic, genotoxic, and neurotoxic effects, further efforts by the scientific community and food producers are needed to identify new ways of reducing its content in food. It appears that the entry into force of regulations establishing maximum limits for acrylamide in various food categories could also contribute to reducing dietary exposure to this compound.

## Figures and Tables

**Figure 1:**
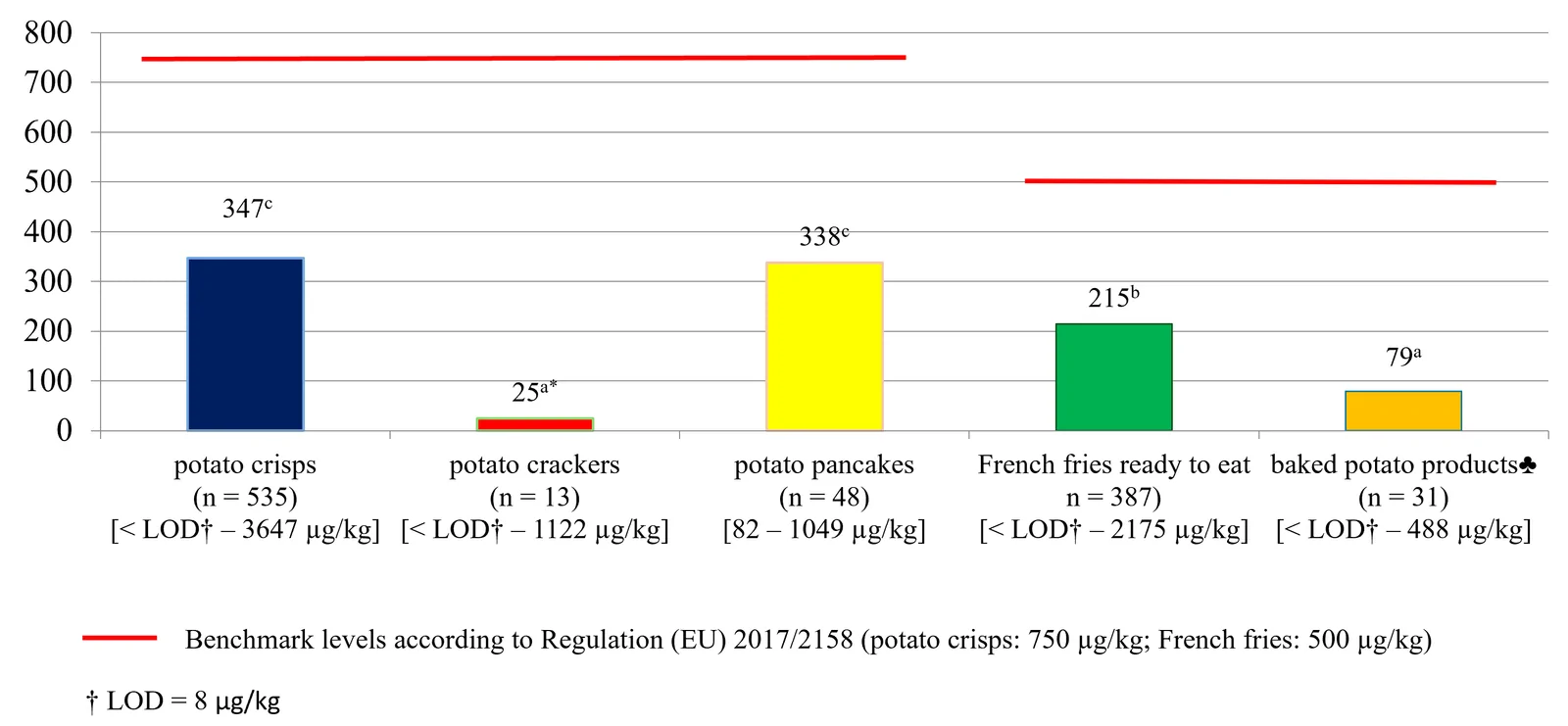
Comparison of acrylamide content in different types of potato products tested in Poland between 2004 and 2025. * Statistically significant difference (*p* < 0.005) between the median acrylamide content over the study period for different groups of potato products. Values marked with different letters are significantly different. ^♣^ Sliced potato (e.g., wedges, potato boats, rounds, etc.) and potato dough products (e.g., balls, cones, etc.).

**Figure 2:**
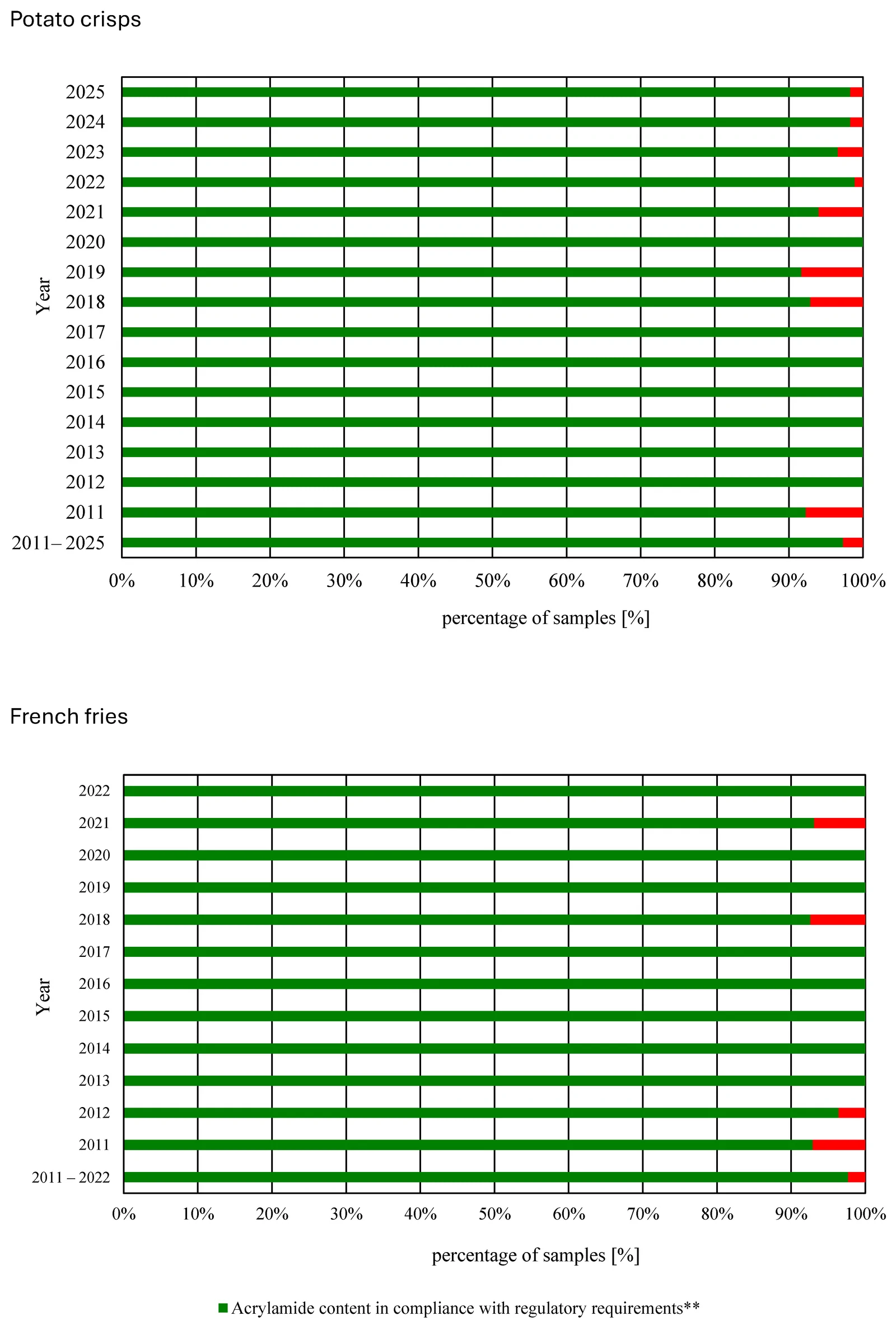
Analysis of potato product samples (potato crisps and French fries) tested in Poland between 2004 and 2025 for compliance with indicative values and benchmark levels for acrylamide. Potato crisps (2011–2025); French fries (2011–2022); ^**^ indicative acrylamide value according to the recommendations of 10 January 2011 [33] and 8 November 2013 (No. 2013/647/EU) [35]—potato crisps: 1000 µg/kg; French fries: 600 µg/kg; benchmark levels for the presence of acrylamide according to Regulation (EU) 2017/2158 [36]: potato crisps: 750 µg/kg; French fries: 500 µg/kg.

**Figure 3:**
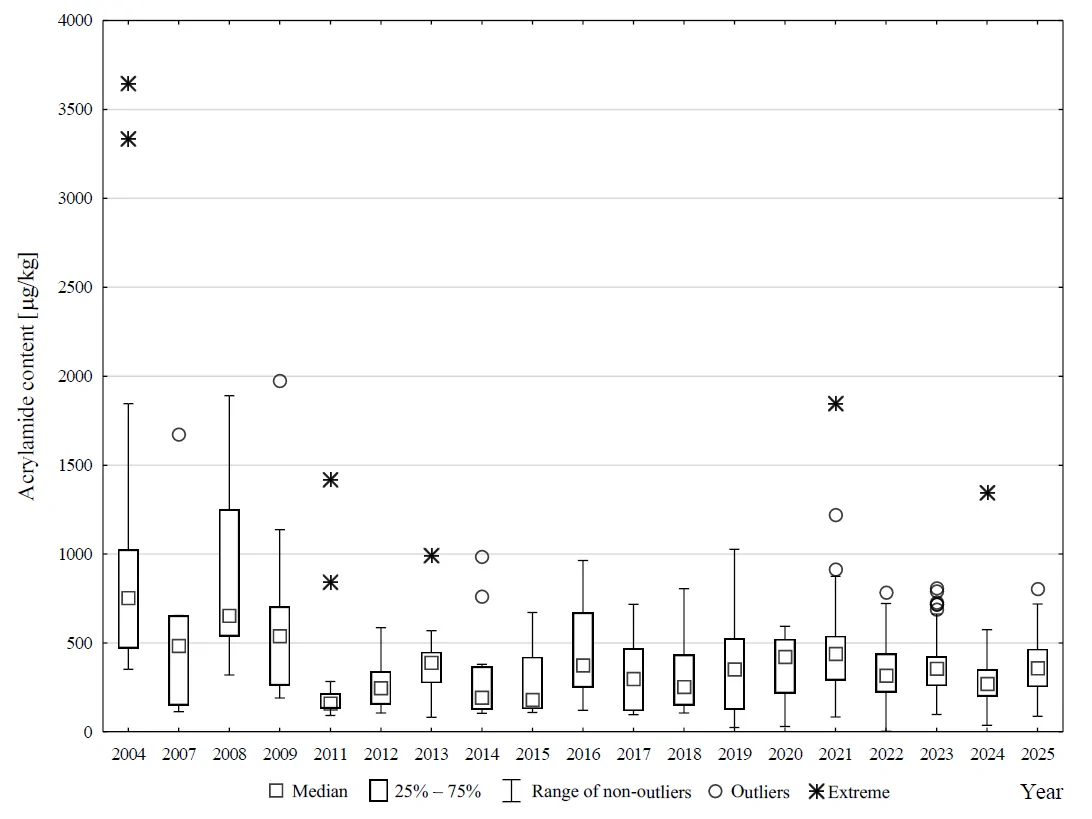
Distribution of acrylamide levels in potato crisps collected from the Polish market between 2004 and 2025.

**Figure 4:**
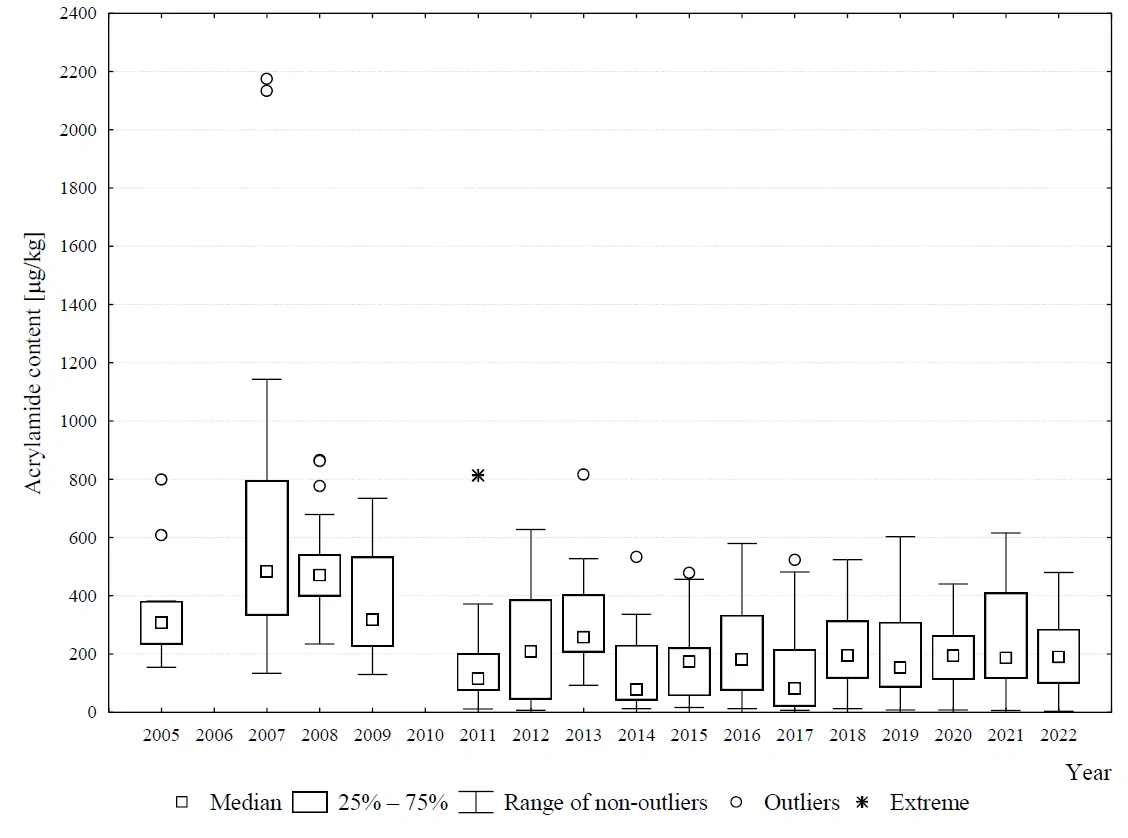
Distribution of acrylamide levels in French fries collected from the Polish market between 2005 and 2022.

**Figure 5:**
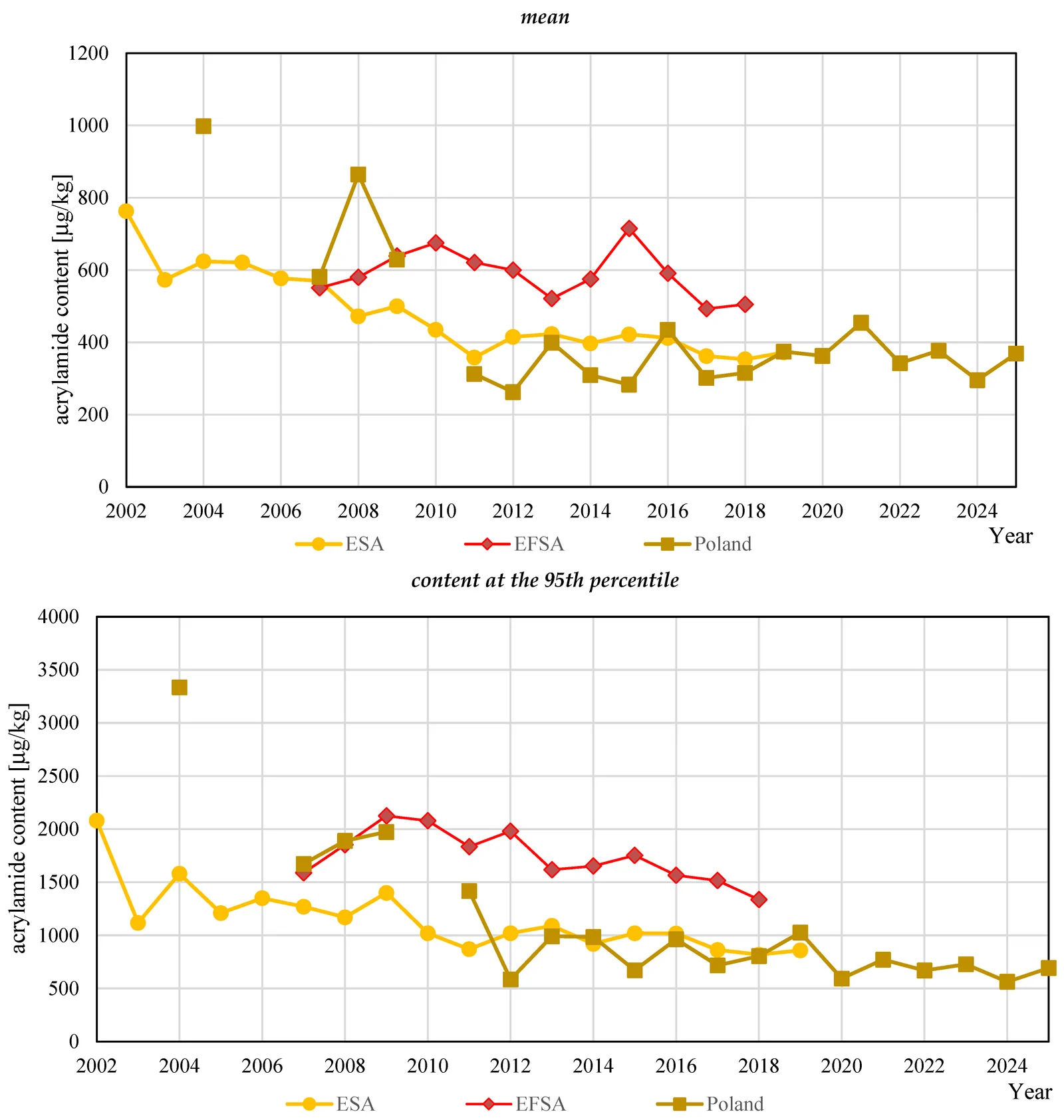
Comparison of acrylamide content in potato crisps in Poland and Europe between 2002 and 2025. Based on Powers et al. [45], EFSA [27,34], Gielecińska & Mojska [39]; Gielecińska & Mojska (unpublished data).

**Figure 6:**
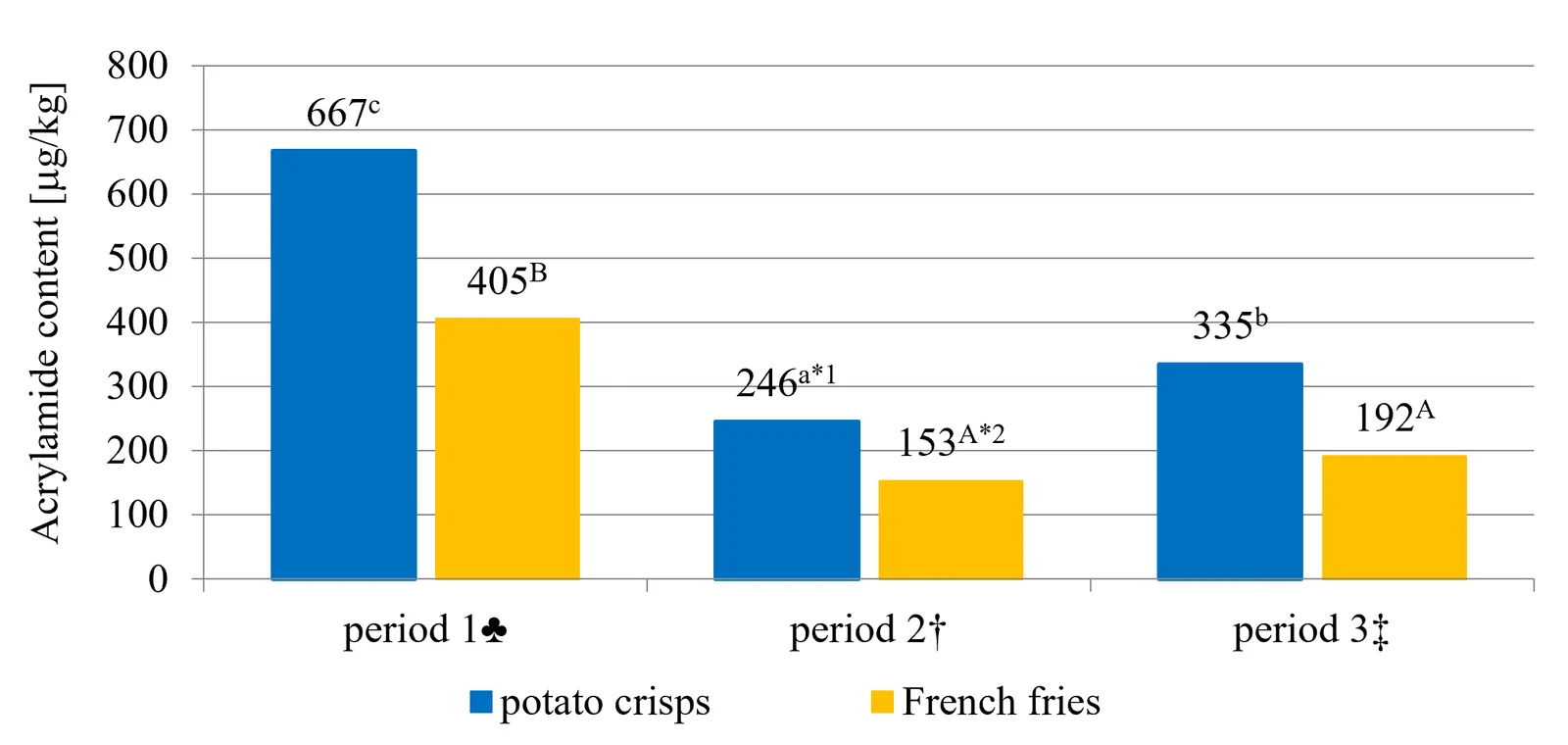
Comparison of acrylamide content in potato crisps and French fries tested in Poland between 2004 and 2025, taking into account the periods before and after the entry into force of legislation establishing indicative values or benchmark levels for acrylamide in food. ^*1^ Statistically significant difference (*p* < 0.001) between the median acrylamide content in potato crisps over the study period for different periods study. Values marked with different letters are significantly different. ^*2^ Statistically significant difference (*p* < 0.001) between the median acrylamide content in French fries over the study period for different periods study. Values marked with different letters are significantly different. ♣ Period 1 [2004–2010 (potato crisps) and 2005–2010 (French fries)]—before the entry into force of legislation establishing indicative values of acrylamide in food. † Period 2 [2011–2017]—Commission Recommendations (of 10 January 2011 [33] and No. 2013/647/EU [35])—indicative value: potato crisps—1000 µg/kg; French fries—600 µg/kg. ‡ Period 3 [2018–2025 (potato crisps) and 2018–2022 (French fries)]—Regulation (EU) 2017/2158 [36]—benchmark levels: potato crisps—750 µg/kg; French fries—500 µg/kg.

**Figure 7:**
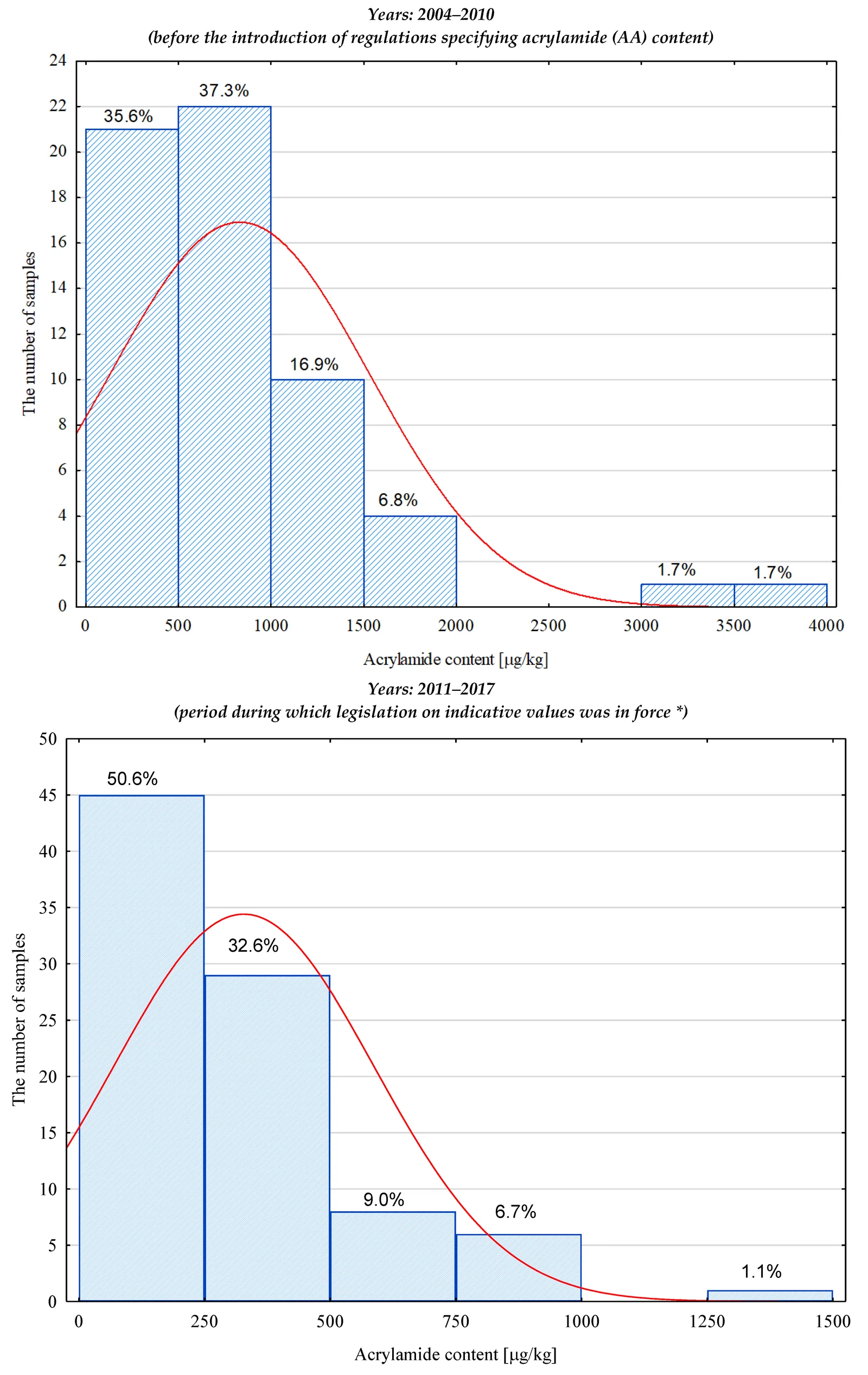

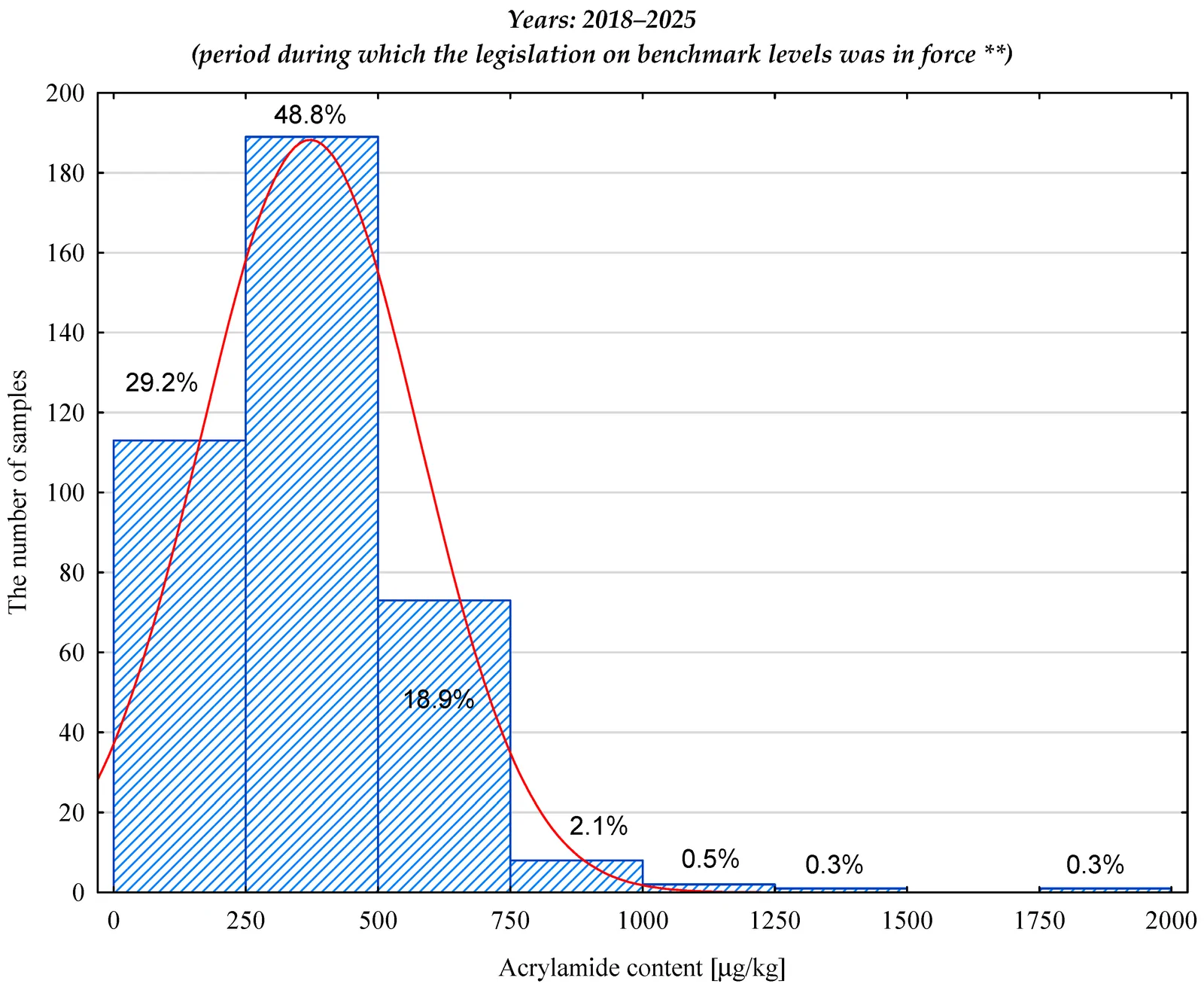
Distribution of the number of potato crisps samples by acrylamide (AA) content. * Commission Recommendations of 10 January 2011 [33]; No. 2013/647/EU [35]. ** Commission Regulation (EU) 2017/2158 [36].

**Figure 8:**
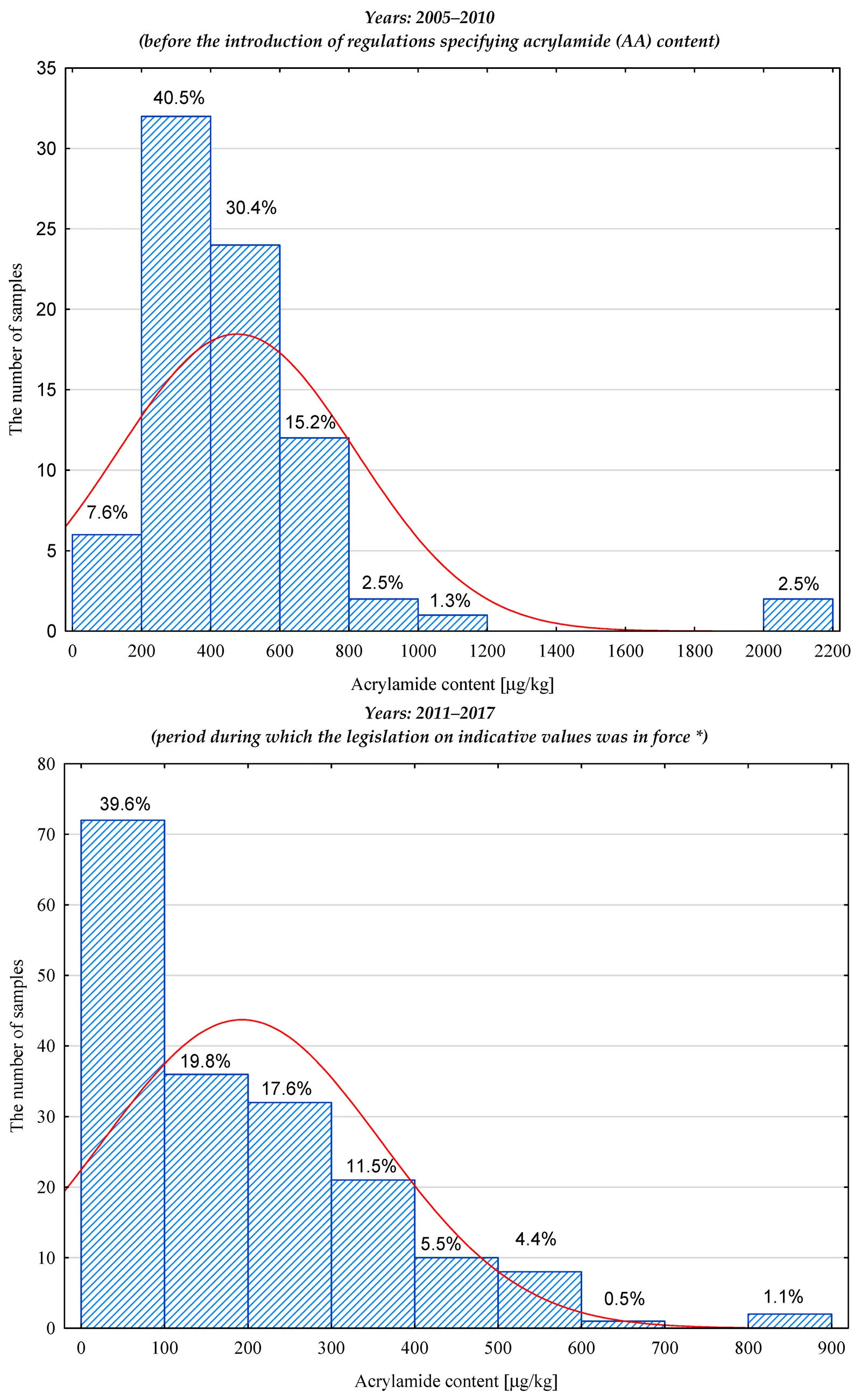

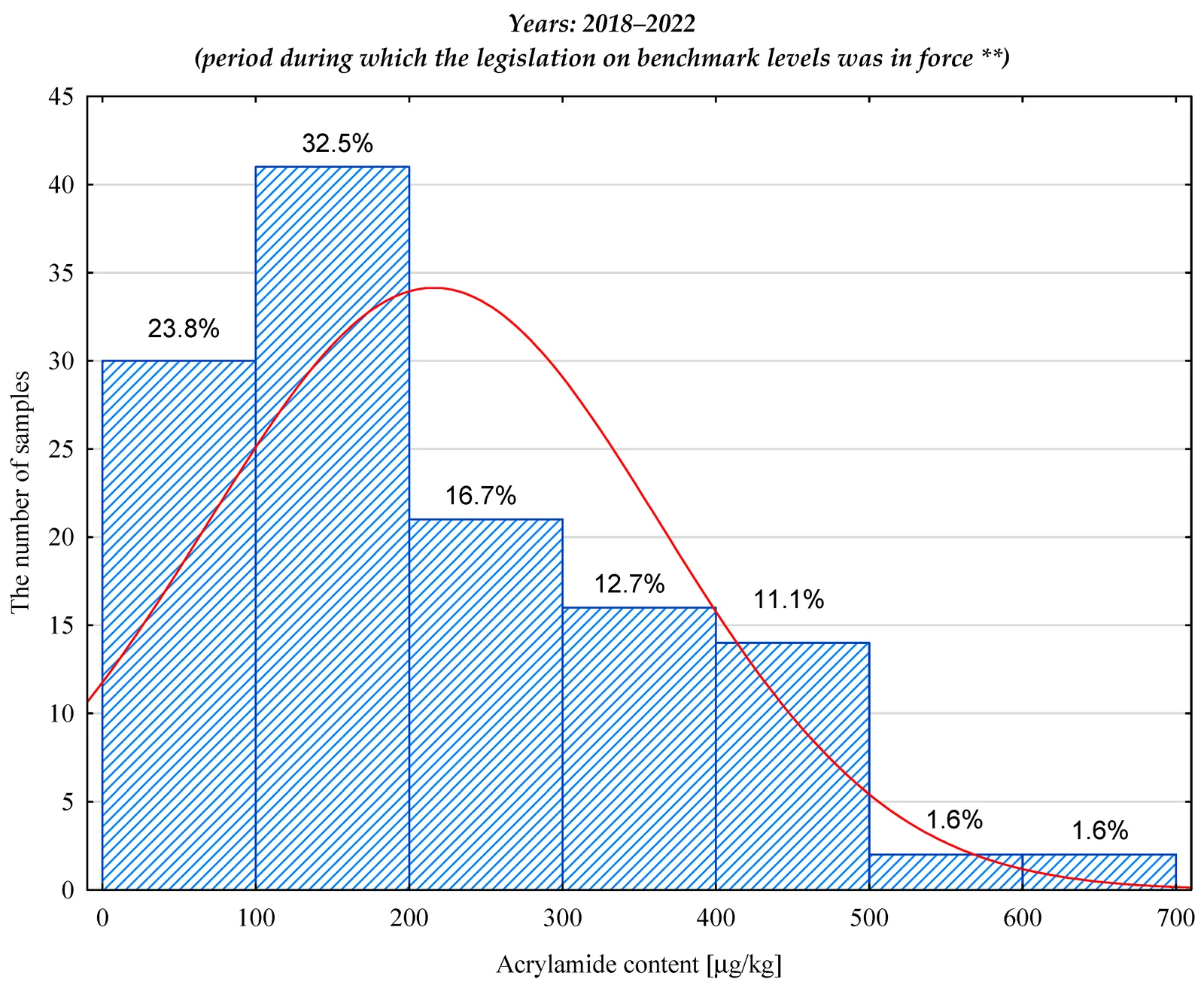
Distribution of the number of French fries samples by acrylamide (AA) content. * Commission Recommendations of 10 January 2011 [33]; No. 2013/647/EU [35]. ** Commission Regulation (EU) 2017/2158 [36].

**Scheme 1:**
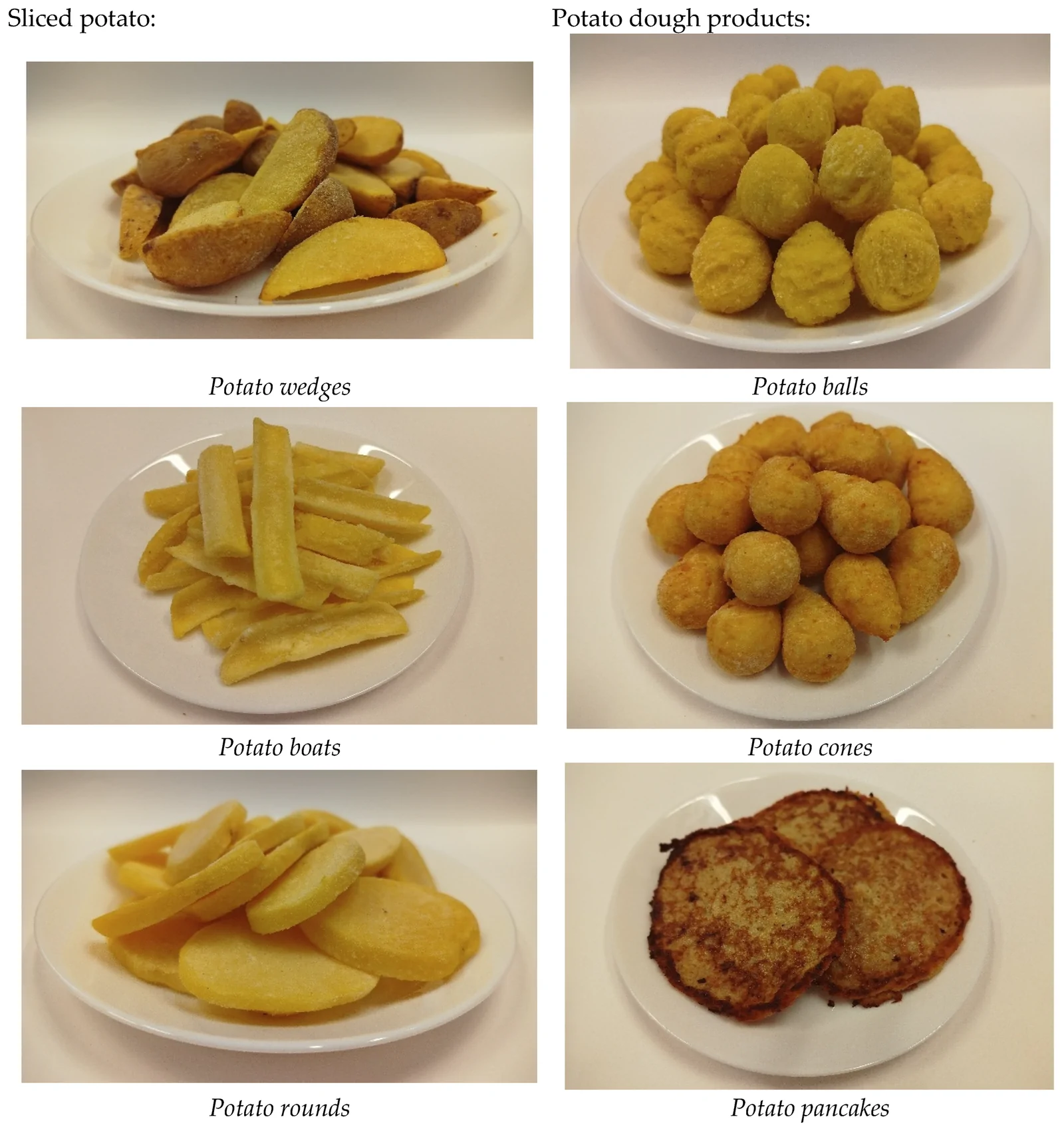
Potato products other than chips and French fries.

**Table 1:** Characteristic of potato product samples analysed as part of monitoring studies conducted in Poland between 2004 and 202.

Product Category According to Commission Regulation (EU) 2017/2158 [36]	Product Group	Sampling Year	Total Number of Samples Analysed	Sampling Location	Characteristics/Type of Sampled Product
– Potato crisps from fresh potatoes and from potato dough – Potato-based crackers – Other potato products from potato dough	Potato crisps	2004–2009 2011–2025	535	retail	ready-to-eat product
	Potato crackers	2015; 2021	13	retail	ready-to-eat product
	Potato pancakes	2014; 2018; 2025	48	catering/retail	ready-to-eat product/frozen semi-finished products intended for further heat treatment at home ^1^
– French fries (ready-to-eat)	French fries	2005–2009 2011–2022	387	catering/retail	ready-to-eat product/frozen semi-finished products intended for further heat treatment at home ^2^
	Sliced potato (e.g., wedges, potato boats, rounds, etc.); potato dough products (e.g., balls, cones, etc.)	2023	31	retail	frozen semi-finished products intended for further heat treatment at home ^3^

^1^ A ready-to-eat product was prepared in the laboratory: it was fried according to the manufacturer’s instructions on the product packaging; ^2^ a ready-to-eat product was prepared in the laboratory: it was heat-treated (deep-fryer; deep-frying) in accordance with the information provided on the product label; and ^3^ a ready-to-eat product was prepared in the laboratory: it was baked in an air fryer with a rotating basket or in a convection oven, in accordance with the manufacturer’s instructions on the product packaging.

## Data Availability

The original contributions presented in this study are included in the article. Further inquiries can be directed to the corresponding author.
